# Characterization of Five *Escherichia coli* Isolates Co-expressing ESBL and MCR-1 Resistance Mechanisms From Different Origins in China

**DOI:** 10.3389/fmicb.2019.01994

**Published:** 2019-08-29

**Authors:** Pei Zhang, Juan Wang, Xinglong Wang, Xue Bai, Jiangang Ma, Ruyi Dang, Yifei Xiong, Séamus Fanning, Li Bai, Zengqi Yang

**Affiliations:** ^1^College of Veterinary Medicine, Northwest A&F University, Xianyang, China; ^2^Guangdong Provincial Key Laboratory of Food, Nutrition and Health, School of Public Health, Sun Yat-sen University, Guangzhou, China; ^3^NHC Key Laboratory of Food Safety Risk Assessment, China National Center for Food Safety Risk Assessment, Beijing, China; ^4^College of Food Science and Engineering, Northwest A&F University, Xianyang, China; ^5^UCD-Centre for Food Safety, School of Public Health, Physiotherapy and Sports Science, University College Dublin, Dublin, Ireland

**Keywords:** ESBL, co-occurrence, conjugative plasmids, *mcr-1* gene, *Escherichia coli*

## Abstract

Present study characterized five *Escherichia coli* co-expressing ESBL and MCR-1 recovered from food, food-producing animals, and companion animals in China. Antimicrobial susceptibility tests, conjugation experiments, and plasmid typing were performed. Whole genome sequencing (WGS) was undertaken for all five isolates using either PacBio RS II or Illumina HiSeq 2500 platforms. The cefotaxime and colistin resistance encoded by *bla*_*CTX–M*_ and *mcr-1* genes, respectively, was transferable by conjugation either together or separately for all five strains. Interestingly, the ESBL and *mcr-1* genes could be co-selected by cefotaxime, while the colistin only selected the *mcr-1*-carrying plasmids during the conjugation experiments. Five *E. coli* sequence types (ST88, ST93, ST602, ST162, and ST457) were detected. Although diverse plasmid profiles were identified, IncI2, IncFIB, and IncFII plasmid types were predominant. These five clonally unrelated isolates harbored the *mcr-1* gene located on similar plasmid backbones, which showed high nucleotide similarity to plasmid pHNSHP45. The *mcr-1* gene can be co-transmitted with *bla*_*CTX–M*_ genes through IncI2 plasmids with or without IS*Apl1* in our study. Characterization of these co-existence ESBL and mcr-1 isolates extends our understanding on the dissemination of these resistance markers among bacteria of diverse origins.

## Introduction

Multidrug resistant (MDR) Enterobacteriaceae isolates have been widely reported in non-clinical environments, from pets and farm animals as well as food, which increases the risk for humans regarding the foodborne or zoonotic transmission routes. The emergence of transferable polymyxin resistance mediated by *mcr-1* represents a threat to the revival of polymyxin E, which has emerged as our last-resort drug for treating carbapenem-resistant Enterobacteriaceae infections (Michalopoulos et al., 2005; Liu et al., 2016; Paterson and Harris, 2016). Since the first plasmid-mediated *mcr-1* gene was reported in members of the Enterobacteriaceae family isolated from humans and food-producing animals in China in late 2015, *mcr-1* has been recognized in over 50 countries/regions covering five continents (Liu et al., 2016; Sun et al., 2018). Several retrospective studies have reported *mcr-1* gene in *Escherichia coli*, *Escherichia albertii*, *Klebsiella pneumonia*, and *Salmonella* species from different geographical locations, with the earliest evidence pointing to its existence dating back to the 1980s (Brennan et al., 2016; Figueiredo et al., 2016; Schwarz and Johnson, 2016; Shen et al., 2016).

Mobile genetic elements (MGE), especially plasmids, are considered to be a key driver of horizontal gene transfer (HGT) of the ten *mcr-1* variants reported to date (Shen et al., 2018). So far, various plasmid replicon types, such as IncI2, IncHI1, IncHI2, IncP, IncFIB and IncX4 have been reported to be associated with HGT of the *mcr* genes (Doumith et al., 2016; Poirel et al., 2016; Zurfluh et al., 2016). It is unusual, but not without precedent, that *mcr-1* has already been found to coexist with both ESBL and *bla*_*NDM–*__1_ (or its variants *bla*_*NDM–*__5_ and *bla*_*NDM–*__9_) on these promiscuous plasmids (Du et al., 2016; Haenni et al., 2016; Li Y. et al., 2018). Moreover, the insertion sequence IS*Apl1* has also been shown to be a key element mediating translocation of *mcr-1* into plasmids of diverse replicon types through formation of a circular intermediate (Li et al., 2017). Notably, the IS*Apl1*-*mcr-1*-cassette was also found to be located on the chromosome, which further supports the hypothesis that IS*Apl1* might be involved in *mcr-1* acquisition. Thus investigation of the MGE, especially plasmids and insertion sequences, is a key component required for a better understanding of the dissemination of *mcr-1* gene and others.

Our study was initiated by the isolation of five cefotaxime and colistin co-resistant *E. coli* cultured originally from 541 isolates taken from different regions in China from 2012 to 2016. In this paper, we reported the characterization of five *E. coli* isolates, with the aims of this study being (i) to determine the co-occurrence of ESBL and MCR-1 markers, (ii) to identify the co-transference mechanisms by dual conjugation assays, (iii) to characterize the *E. coli* by WGS, and (iv) to identify the plasmid backbones and genetic environments containing the *mcr-1* genes.

## Materials and Methods

### Bacterial Collection

In this study, five *mcr-1* positive and cefotaxime resistant isolates were detected among 265 *E. coli* cultured from retail food (*n* = 2), 69 *E. coli* isolates from sick companion dogs (*n* = 1), 93 *E. coli* isolates from pigs (*n* = 1), and 114 *E. coli* isolates from poultry (*n* = 1). All isolates were collected in geographically distinct regions located in Beijing, Tianjin, Hangzhou, Guiyang, Chongqing cities during 2012–2016 in China. The species of all isolates were determined by 16S rDNA sequencing. Primers targeting the *mcr-1* gene were described previously used to screen the studied isolates (Liu et al., 2016).

### Antimicrobial Susceptibility Testing and Detection of Resistance Genes

The minimum inhibitory concentration (MIC) of colistin (Sigma, St Louis, MO, United States) was determined by broth dilution method according to the European Committee on Antimicrobial Susceptibility Testing (EUCAST)^1^. In addition, all isolates were tested for their susceptibility to a panel of antimicrobial compounds by disk diffusion, following recommendations of the Clinical and Laboratory Standards Institute (Clinical and Laboratory Standards Institute [CLSI], 2016). Reference strain *E. coli* ATCC^TM^ 25922 served as a quality control. Thereafter, the β-lactamase-encoding genes belonging to the *bla*_*TEM*_, *bla*_*CTX–M*_, *bla*_*GES*_, *bla*_*PER*_, and *bla*_*VEB*_ families were screened by PCR (Dallenne et al., 2010).

### Conjugation Based Mating Experiments and Verification

Five ESBL- and *mcr-1-*positive isolates were analyzed individually, for their ability to transfer colistin/cefotaxime resistance to a rifampicin-resistant, plasmid-free *E. coli* recipient (26R 793) (Wang J. et al., 2016a). Conjugation experiments were carried out in triplicate using a broth mating protocol as described previously (Wang J. et al., 2013). Transconjugants were selected on plates containing 2 μg/mL colistin with 100 μg/mL rifampicin or 4 μg/mL cefotaxime with 100 μg/mL rifampicin. Transfer frequencies were calculated per donor cell. Antimicrobial susceptibility tests were performed to confirm the plasmid transfer as described above, followed by S1-nuclease pulsed-field gel electrophoresis (PFGE) and PCR-based replicon typing (PBRT) to test which plasmids and resistance markers were transferred.

### Plasmid Profiling and PCR-Based Replicon Typing

S1-nuclease-digested (Promega, Madison, WI, United States) linearized plasmid DNAs from the five ESBL and MCR-1 positive isolates and their transconjugants were further examined by PFGE for the detection and size determination of large plasmids as described previously (Barton et al., 1995). The approximate molecular mass of plasmids was determined by comparing with *E. coli* 39R 861 (Macrina et al., 1978). The isolates were also examined for the presence of 18 replicon types by PBRT (Carattoli et al., 2005).

### Whole Genome Sequencing and Phylogenetic Analysis

To assess the genomic background of the five isolates, whole genome sequencing (WGS) was performed using the PacBio RS II system (for CQ9 isolate) or the Illumina HiSeq 2500 platform (Illumina, San Diego, CA, United States) (for F81, F89, Dog1, and CY69 isolates). Reads from Illumina platform were assembled *de novo* using SOAPdenovo^2^. Annotation of the genomes was performed using RAST^3^, BLASTn and BLASTp^4^ programs. The ORF Finder program^5^ was also used to identify features. Standard methods were used to annotate the serotype, multidrug resistance genes, and plasmid incompatibility types based on the WGS data by CGE platforms^6^. Sequence comparison and map generation were performed using BLAST and Easyfig version 2.1.

### Nucleotide Accession Numbers

Sequences were deposited in GenBank under accession numbers: CQ9 genome (CP031546), pCQ9-1 (CP031547), pCQ9-2 (CP031548), pCQ9-3 (CP031549) and pCQ9-4 (CP031550), F81 (QXGP01000001 to QXGP01000155), F89 (QXGO01000001 to QXGO01000093), Dog1 (QUON01000001 to QUON01000107), and CY69 (QUOM01000001 to QUOM01000089).

## Results and Discussion

### Bacterial Isolation and Molecular Characterization

The five (∼1%) cefotaxime- and colistin-resistant isolates were identified from totally 541 *E. coli* strains from animal and food sources of different geographical regions in China from 2012 to 2016 (Table 1). The prevalence is much lower than the data reported earlier (Li et al., 2017; Shen et al., 2018; Wu C. et al., 2018), and one possible reason for this could be the diverse sources and different geographical origins of the samples. All five isolates were sub-typed by MLST and the corresponding serotypes identified *in silico* following analysis of the WGS data. These *E. coli* isolates were assigned to five different sequence types/serotypes: ST88 (O116:H11), ST93 (O5:H10), ST602 (O18:H11), ST162 (ST469 complex, Ont:H19), ST457 (O3:H25), and suggesting genetic diversity among these isolates. The diversity of sequence-type clades of cefotaxime and colistin co-expressing strains further confirms the wide distribution of ESBLs- and *mcr*-carrying *E. coli* and that corresponding resistance is mediated mainly by plasmids rather than adapted dominant bacterial clones (Shen et al., 2018).

**TABLE 1:** Features of *mcr-1* positive *E. coli* strains include the species, serotype, as well as year, original, and MLST are also shown.

**Strains**	**Original**	**Year**	**Serotype**	**MLST**	**Plasmid finder**	**MDR genes**	**Sequencing platform**	**Accession number**
CQ9	Pig	2012	O116:H11	ST88	IncHI2; IncHI2A; IncFIC(FII); IncFIB; IncX1; IncFII(29); IncA/C2; and IncI2	*bla*_*CTX–M–55*_, ***mcr-1*,** *bla*_*CMY–2*_, *strA/B*, *aadA2*, *aadA1*, *aph(3’)-Ia*, *erm(B)*, *mph(A)*, *floR*, *cmlA1*, *sul1*, *sul2*, *sul3*, *tet*(A), and *dfrA12*	PacBio RS II system	CP031546
F81	Retail food	2014	O5:H10	ST93	IncFIB-69; IncFII-154; IncFIC(FII)-137; IncR-51; and IncI2-31	*bla*_*CTX–M–55*_, ***mcr-1*,** *strA/B*, *aph(3’)-Ia*, *aadA2*, *bla*_*TEM–1B*_, *qnrS1*, *oqxA/B*, *fosA*, *mph(A)*, *floR*, *sul1*, *sul2*, *tet*(A), and *dfrA12*	Illumina MiSeq	QXGP01000001 to QXGP01000155
F89	Retail food	2014	O18:H11	ST602	IncFIB-47; IncFIC-32; and IncI2-21	*bla*_*CTX–M–64*_, ***mcr-1*,** *strA/B*, *oqxA/B*, *floR*, *sul2*, *tet*(A), and *dfrA17*	Illumina MiSeq	QXGO01000001 to QXGO01000093
Dog1	Dog	2016	Ont:H19	ST162 (ST469 Cplx)	IncFIB-55; IncFII-30; IncI2-27	*bla*_*CTX–M–24*_, ***mcr-1*,** *strA/B*, *aac(3)-IId*, *aph(3’)-Ia*, *bla*_*TEM–1B*_, *mph(A)*, *floR*, *sul2*, *tet*(A)	Illumina MiSeq	QUON01000001 to QUON01000107
CY69	Poultry	2016	O3:H25	ST457	IncFIB-47; IncFII-54; and IncI2-27	*bla*_*CTX–M–27*_, ***mcr-1*,** *aadA2*, *aph(3’)-Ia*, *strA/B*, *rmtB*, *bla*_*TEM–1B*_, *fosA*, *mph(A)*, *erm(B)*, *floR*, *sul1*, *sul2 tet*(A), and *dfrA12*	Illumina MiSeq	QUOM01000001 to QUOM01000089

**FIGURE 1:**
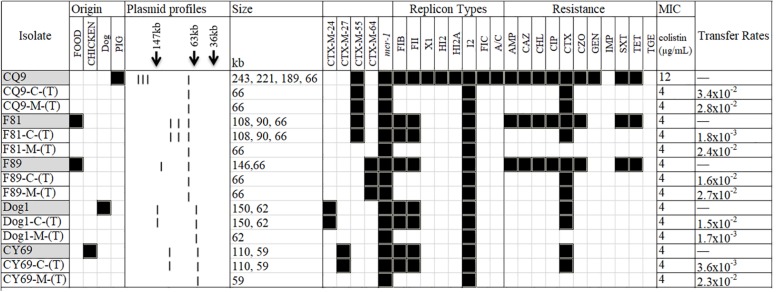
A heat-map showing the comparison of the five *Escherichia coli* donors and the resultant transconjugants, characterized on the basis of their plasmid profiles according to the S1-PFGE results; ESBL- and *mcr-1*-markers identified by PCR; antimicrobial resistance profile; and plasmid replicon type(s). The MIC for colistin in each group combination is also shown. (T) in the first column typifies the transconjugant. C and M in the first column typifies cefotaxime and colistin, respectively. Black and white squares denote the presence and absence, respectively, of a particular feature. Antimicrobial compounds are abbreviated as follows: AMP, ampicillin; CAZ, ceftazidime; CHL, chloramphenicol; CIP, ciprofloxacin; CTX, cefotaxime; CZO, cefazolin; GEN, gentamicin; IMP, imipenem; SXT, trimethoprim/sulfamethoxazole; TET, tetracycline; TGE, tigecycline.

S1-nuclease plasmid analysis revealed that all five isolates contained high molecular weight plasmids (ranging from approximately 50- to ∼250-kb); the CQ9 isolate possessed four plasmids, F81 possessed three plasmids, and each of the remaining three isolates each possessed two plasmids (for F89, Dog1, and CY69). Although heterogeneity among the plasmid profiles was a common feature noted, all isolates carried one plasmid of approximately 60-kb in size (Figure 1). Notably, the 60-kb plasmids from all five strains were transferred by conjugation under laboratory conditions after selection by either colistin or cefotaxime.

PBRT and Plasmid finder showed that all isolates possessed at least three plasmid replicons: including the IncI2, IncFIB and IncFII replicon types, which were considered to be the epidemic resistance plasmid replicon types bearing the greatest variety of resistance genes in Enterobacteriaceae (Carattoli, 2011; Rozwandowicz et al., 2018). One isolate, F81 from retail food also carried an IncR plasmid, which is increasingly described as a reservoir of multidrug resistance (Compain et al., 2014; Kocsis et al., 2016). Consistent with its plasmid profile, the isolate CQ9 of porcine origin possessed a broader plasmid incompatibility profile, in addition to the ones described above, which also contained the IncHI2, IncX1, and IncA/C2 type plasmids. A summary of these features along with the corresponding antimicrobial resistance profiles for all 5 isolates and their transconjugants is shown in Figure 1 and Table 1.

### Antibiotic Resistance and Associated Determinants of the ESBL and MCR-1 Co-producing Isolates

Phenotypic characterization of antibiotic resistance indicated that all the five cefotaxime and colistin co-expressing isolates, also expressed a multidrug-resistant phenotype (resistance to more than two antibiotic classes) and resistance to chloramphenicol, ciprofloxacin and tetracycline in addition to the colistin and β-lactam antibiotics were recorded (Figure 1). In contrast, all isolates were susceptible to aztreonam, imipenem and cefepime. The MICs of colistin for the donor strains and their transconjugants were between 4 and 8 μg/mL (Figure 1).

Resfinder, using WGS, identified the corresponding molecular mechanisms for antimicrobial resistance phenotypes involved in all five isolates. The genes associated with the colistin resistance were confirmed as *mcr-1* gene in all cases. All strains were confirmed as ESBL-producing with different *bla*_*CTX–M*_ (*n* = 5, *bla*_*CTX–M–*__24__/__27__/__55__/__55__/__64_) and the same *bla*_*TEM–*__1__*B*_ (*n* = 3) genes (Table 1). In addition, CQ9 was the only isolate harbored the widest distribution of the *ampC*-like gene *bla*_*CMY–*__2_. With the exception of the MDR genes above, the strains were also found to harbor multiple resistance elements, including but not limited to *oqxAB, floR*, *fosA*, *dfrA*, *erm*(B) and *mph*(A), which were consistent with the antimicrobial resistance phenotypes in the study (Table 1).

### Dual Conjugation Experiments With Antibiotic Selections

To better address whether the co-transfer of the ESBL and *mcr-1* genes of the isolates could occur, conjugation assays were carried out in triplicate. Five isolates transferred their cefotaxime resistance determinant to a rifampicin-resistant, plasmid-free *E. coli* 26R 793 recipient with transfer rates ranging from 1.8 × 10^–3^ (for F81) to 3.4 × 10^–2^ (for CQ9) transconjugants per donor cell (Figure 1). The *bla*_*CTX–M*_ genes transferred *via* conjugation were subsequently confirmed by PCR. Interestingly, the five transconjugants selected by cefotaxime and rifampicin were also resistant to colistin.

Furthermore, to assess the transformation of the colistin-resistant phenotype for each donor isolate, an independent conjugation experiment was set up using colistin and rifampicin for selection was also performed. It is notable that all five isolates transferred the colistin resistance marker to the recipient with transfer rates ranging from 1.7 × 10^–3^ (for Dog1) to 2.8 × 10^–2^ (for CQ9) (Figure 1). However, it is very interesting to note that only two transconjugants from CQ9 and F89 were resistant to cefotaxime.

Conjugation studies and plasmid profile analysis of all transconjugants showed that the five donor isolates possessed one plasmid of approximately 60-kb. Without exception, these plasmids were all transferred to the recipients. Following PBRT, the transferrable plasmids were identified as belonging to the IncI2 replicon type. Three isolates (F81, Dog1, and CY69) were identified wherein all high molecular weight plasmids were transferred *via* conjugation under the selection by cefotaxime whilst the other two (CQ9 and F89) transferred a single IncI2-type plasmid under the selection pressure of either cefotaxime or colistin (Figure 1).

In our study, it is very interesting to note that all of the *mcr-1* encoding plasmids can be transferred under the selection pressure of colistin and cefotaxime, but the *bla*_*CTX–M*_ encoding plasmids can only be transferred under the selection-pressure of cefotaxime. Cefotaxime could co-select the ESBL and *mcr-1* genes, while the use of colistin only selected the *mcr-1*-carrying plasmids. It is not clear why and how the addition of the two selection antibiotics worked. However, a previous longitudinal study showed that the prevalence of *mcr-1* was higher in the ESBL-positive *E. coli* than in the non-ESBL *E. coli*, and the rapid rising in ESBL prevalence apparently also increased the selective pressure of colistin resistance (Wu C. et al., 2018). Based on these observations, it is tempting to speculate that an antibiotic can affect the overall conjugation dynamics by modulating the conjugation efficiency, serving as a selection force, in itself, that acts on the population dynamics after conjugation, or both (Lopatkin et al., 2016).

### Co-occurrence and Co-transference of *bla*_*CTX–M*_ and *mcr-1* Genes

Subsequent plasmid profile analysis showed that all transconjugants contained 1∼3 detectable large molecular-weight plasmids ranging from 58- to 150-kb in size (Figure 1). Two plasmids were identified from the transconjugants of Dog1-C-(T) and CY69-C-(T), but only one plasmid from Dog1-M-(T) and CY69-M-(T) were selected by colistin with rifampicin. Further analysis of the MDR markers indicated the *mcr-1* gene and *bla*_*CTX–M*_ in the Dog1 and CY69 isolates were located on two separate plasmids. There were three large plasmids identified from F81. All three were transferrable under the selection of cefotaxime with rifampicin, however, only the smallest plasmid (58-kb in length and *mcr-1*-harboring) was transferred to the recipient under the selection pressure of colistin with rifampicin (Figure 1). In addition to colistin resistance, resistance to several antimicrobial compounds such as ampicillin, chloramphenicol, and cefotaxime were also transferred to the recipient, suggesting that these markers are genetically linked (Figure 1).

One plasmid (66-kb in size) from the strain CQ9 was transferred to the recipients in the two independent conjugation experiments. A similar scenario was noticed in F89, wherein a single plasmid of 63-kb was detected from the transconjugants following selection using cefotaxime with rifampicin and colistin with rifampicin. The cefotaxime resistance determinant was confirmed as *bla*_*CTX–M–*__55_ by PCR in CQ9-C-(T) (the corresponding transconjugant selected by cefotaxime with rifampicin) and CQ9-M-(T) (the corresponding transconjugant selected by colistin with rifampicin) and as *bla*_*CTX–M–*__64_ in F89-C-(T) and F89-M-(T), which showed the donor strains of the two had the *mcr-1* gene and *bla*_*CTX–M*_ gene co-transferred on the same plasmid (Figure 1). Following PBRT and WGS data analysis, the transferrable and *bla*_*CTX–M*_-harboring plasmids were identified as belonging to the IncI2 replicon type.

It has been shown that the ESBL and *mcr-1* genes could be co-transferred by one or more types of conjugative plasmid, which could allow their efficient dissemination among bacteria (McGann et al., 2016; Sun et al., 2016, 2017; Wu C. et al., 2018). In our study, two isolates contained the *mcr-1* gene along with a *bla*_*CTX–M*_ gene (*bla*_*CTX–M–*__55_ gene from CQ9 and the *bla*_*CTX–M–*__64_ gene from F89) and these were co-transferred on the same plasmid, which was confirmed by the conjugation experiments and the sequencing data. In the rest three isolates the ESBL (*bla*_*CTX–M–*__24__/__27__/__55_) and *mcr-1* genes were located on two separate plasmids. Such phenomena may contribute to the co-selection of antibiotic resistance carried by multiple types of plasmids and caused by improper combination usage of antibiotics. For the *mcr-1*-positive transconjugants in the study, the sizes of plasmids observed were approximately 60-kb and all belonged to IncI2 which is consistent with the previous study of the predominant association of *mcr-1* with narrow-host types of plasmids: IncI2, IncHI2, and IncX4 (Li et al., 2017). It has been proposed the *mcr-1* bearing IncI2 plasmids possess a fitness advantage when compared to other replicon types containing this marker (Wu R. et al., 2018). Moreover, the acquisition of IncI2-type plasmid was more beneficial for host *E. coli* isolate than either IncHI2 or IncX4 plasmid (Wu R. et al., 2018). Thus, the fitness advantage and co-transference/co-selection of *mcr-1* and other antimicrobial resistance genes may further explain the fact that the most reported *mcr-1*-harboring plasmids primarily belong to the IncI2 plasmid types.

### Molecular Characterization and Comparative Analysis of Plasmid Backbones

Genomic DNA (gDNA) purified from the five *mcr-1*-positive strains were subjected to sequencing using either the Illumina platform or the Pacbio RS II system. DNA of CQ9 isolated from the porcine sample in 2012 in Chongqing, was subjected to PacBio sequencing to obtain the complete genome, including, the sequences of all four plasmids (Table 2). From this isolate, the *mcr-1* and *bla*_*CTX–M–*__55_ co-harboring plasmid namely pCQ9-4 (CP031550), was found to be 66,947 bp in size and exhibited a high identity (99%) with plasmid pA31-12 isolated from a chicken isolate in Guangzhou in 2012 which highlighted the fact that these ESBL and MCR containing plasmids have been in existence and ecologically fit in different niches (Sun et al., 2016). Two of the other three complete sequences of the plasmids with large sizes derived from CQ9 had multiple resistance elements which belonged to IncHI2 and IncA/C2 (Table 2).

**TABLE 2:** Summary of the features associated with all 4 sequenced plasmids in CQ9 isolate.

**Plasmid number**	**Size (bp)**	**Inc type(s)**	**Antibiotic resistance genes**	**Accession number**
pCQ9-1	243,642	IncHI2 and IncHI2A	*sul1* and *tet*(A)	CP031547
pCQ9-2	221,041	IncFIC(FII), IncFIB, IncX1, and IncFII(29)	None	CP031548
pCQ9-3	189,020	IncA/C2	*bla*_*CMY–2*_, *strA/B*, *aadA2*, *aadA1*, *aph(3’)-Ia*, *erm(B)*, *mph(A)*, *floR*, *cmlA1*, *sul2*, *sul3*, *tet*(A), and *dfrA12*	CP031549
pCQ9-4	66,945	IncI2	*mcr-1* and *bla*_*CTX–M–55*_	CP031550

*Features include the source and size, antibiotic resistance genes, as well as plasmid Inc., types and sizes of transmissible plasmids.*

**FIGURE 2:**
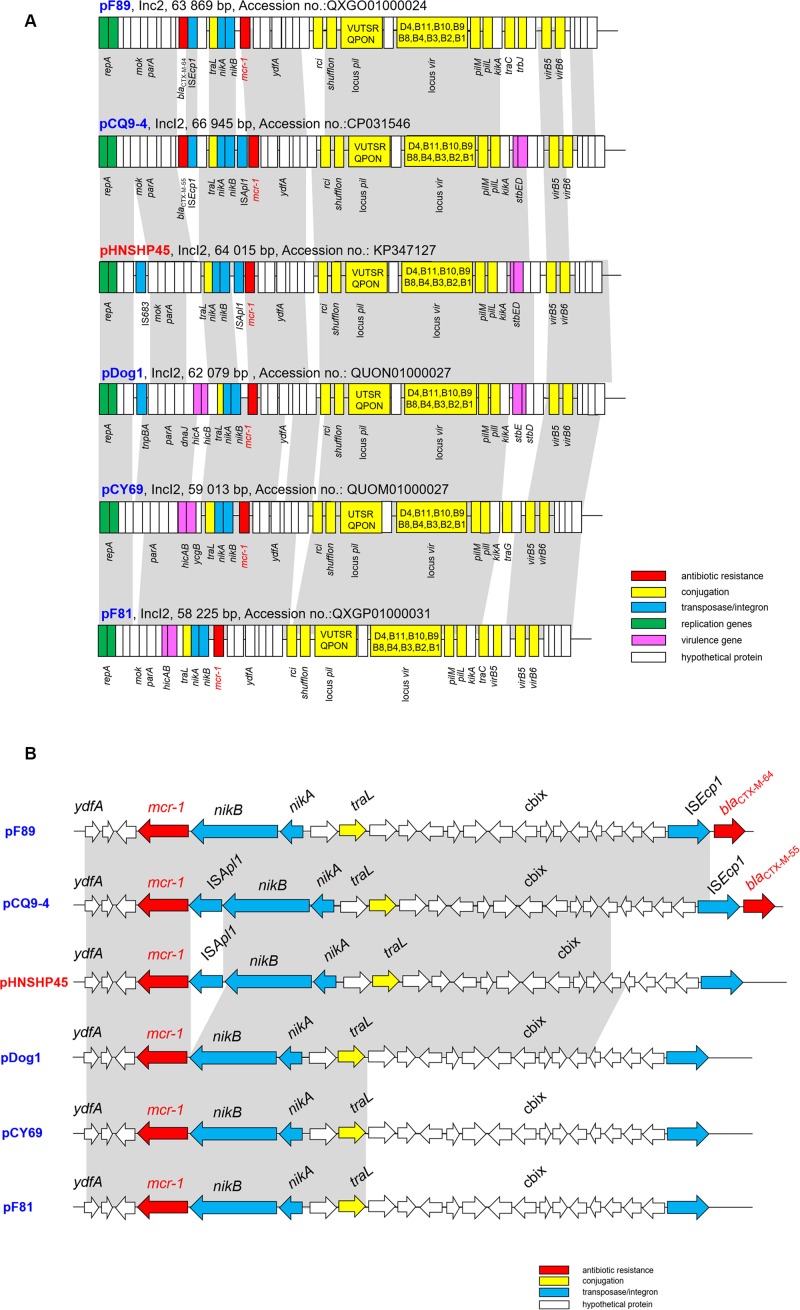
**(A)** Major structural features of the *mcr-1*-harboring IncI2 plasmids in this study in comparison with the reference plasmid pHNSHP45 (Accession number KP347127). **(B)** Comparative schematic representation of the flanking regions of the *mcr-1* genes in IncI2 plasmids. Areas shades in gray indicate homologies in the corresponding genetic environment on each plasmid. The ORFs are shown as boxes or arrows. Antibiotic resistance-encoding genes are indicated in red boxes/arrows. The individual conjugation-related genes are indicated with yellow boxes/arrows and the corresponding genes are indicated with capital letters inside the yellow boxes. Blue boxes/arrows denote transposon- and integron-associated genes. The putative virulence-related genes are indicated by violet boxes. The replication-associated genes are shown in green. White boxes/arrows indicate hypothetical proteins or mobile element proteins [The figures are not drawn to scale].

Complete plasmid sequences were not available for the strains sequenced using the Illumina platform, for which only the assembly scaffolds were generated. Nonetheless, the Illumina contigs of IncI2-typed conjugative plasmids recovered from F81, F89, Dog1, and CY69 isolates were obtained and compared to plasmid pHNSHP45, with results showing that these conjugative plasmids displayed high degrees of sequence homology to pHNSHP45 (Figure 2A). WGS data revealed the genetic context of the plasmids carrying *mcr-1* in this study, had a typical plasmid backbone responsible for plasmid replication, maintenance, and transfer (Figure 2A).

When it comes to the genetic environments of the *mcr-1* genes, none of the isolates contained Tn*6330* (IS*Apl1*-*mcr-1*-*orf*-IS*Apl1*), a structure which could generate a circular intermediate mostly found in IncHI2 *mcr-1* plasmids (Li et al., 2017) and chromosomes (Li R. et al., 2018). Further analysis of the sequencing data obtained separately identified a 1,626-bp contig that contained the *mcr-1*-encoding gene. Interestingly, only one of the five plasmids (for pCQ9-4) was found to contain an IS*Apl1*-*mcr-1* cassette identical to that of plasmid pHNSHP45, whereas in the other four plasmids the *mcr-1*-*pap* cassette was inserted in a conserved position (i.e., downstream of the *nikB* locus) and was not associated with any flanking mobile element (e.g., IS*Apl1*) (Figure 2B). Furthermore, a similar hypothetical protein coding sequence was detected downstream of *mcr-1* in all plasmids, which aligned closely with phosphatidic acid phosphatase-related protein (Pap2) found in *E. coli* (85% identity, GenBank accession number WP_104692553.1). The pCQ9-4 contains a complete IS*Apl1* insertion sequence which has its own IRL (terminal inverted repeat left) and IRR (terminal inverted repeat right), an IRR-like sequence located in pap2 (Wang Q. et al., 2016b). Although the IS*Apl1* cassette was not detected in another four plasmids, partial difference IRR-like sequences were still located in pap2 (Figure 2B).

The same situation has been found in Switzerland in that most of the sequenced *mcr-1* harboring plasmids were lacking the IS*Apl1* element (Zurfluh et al., 2017), which is a key element mediating translocation of *mcr-1* into various plasmid backbones (McGann et al., 2016) and chromosomes (Li R. et al., 2018). It seems likely that complex dissemination of *mcr*-like genes relies on the diffusion of promiscuous plasmids, rather than on the clonal expansion of *mcr-1*-bearing bacteria. Although the prevalence of particular plasmids may vary depending on the source and geographical site, they have been increasingly isolated from bacteria of human, animal and environmental origin, highlighting the frequent exchange of genetic material between different niches.

## Conclusion

In this study, the co-occurrence of plasmid-mediated ESBL and *mcr-1* mechanisms in *E. coli* could be traced back to 2012 and the different MLSTs and diverse *bla*_*ESBL*_ genes in the isolates with different origins further supported that the diversified transfer mechanisms of ESBL and *mcr-1* were present in *E. coli*. Moreover, our findings detected the different efficacy between cefotaxime and colistin during the conjugation experiments. When the ESBL and *mcr-1* genes were harbored by separate plasmids in one isolate, cefotaxime could co-select both of the ESBL and *mcr-1* genes, while the usage of colistin only selected the *mcr-1*-carrying plasmids. This observation may provide insights into one distinct manner of their dissemination; hence, further studies are needed to estimate the effects of antibiotic-mediated selection. Although colistin was banned for use in food animals in China, the preservation of ESBL and *mcr-1* genes through co-selection with other antibiotics and the co-localization on a conjugative plasmid may accelerate the dissemination of both genes by HGT, which insinuated that we should pay more attention to monitoring the prevalence of plasmid mediated MDR genes.

## Data Availability

The datasets generated for this study can be found in the Sequences were deposited in GenBank under the accession numbers: CQ9 genome (CP031546), pCQ9-1 (CP031547), pCQ9-2 (CP031548), pCQ9-3 (CP031549) and pCQ9-4 (CP031550), F81 (QXGP01000001 to QXGP01000155), F89 (QXGO01000001 to QXGO01000093), Dog1 (QUON01000001 to QUON01000107), and CY69 (QUOM01000001 to QUOM01000089).

## Ethics Statement

The protocol in this study was approved by the Committee on the Ethics of Animal Care and Use of National Research Center for Veterinary Medicine (Permit 20160313088). All animal works were carried out in accordance with the recommendation of ethical guidelines of the Animal Care and Use Committee of Northwest A&F University. Individual written informed consent for the use of samples was obtained from all the animal owners and veterinarians.

## Author Contributions

PZ, JW, LB, and ZY conceived and designed the study. XW, RD, XB, and YX acquired the data. PZ, JW, JM, and LB drafted the manuscript. SF critically revised the manuscript.

## Conflict of Interest Statement

The authors declare that the research was conducted in the absence of any commercial or financial relationships that could be construed as a potential conflict of interest.

## Footnotes

1 http://www.eucast.org; accessed March 22, 2016.

2 https://www.animalgenome.org/bioinfo/resources/manuals/SOAP.html

3 http://rast.nmpdr.org

4 http://blast.ncbi.nlm.nih.gov/Blast.cgi

5 http://www.ncbi.nlm.nih.gov/orffinder

6 https://cge.cbs.dtu.dk/services/PlasmidFinder/
